# Shining New Light on the Structural Determinants of Cardiac Couplon Function: Insights From Ten Years of Nanoscale Microscopy

**DOI:** 10.3389/fphys.2018.01472

**Published:** 2018-10-22

**Authors:** Izzy Jayasinghe, Alexander H. Clowsley, Oscar de Langen, Sonali S. Sali, David J. Crossman, Christian Soeller

**Affiliations:** ^1^Faculty of Biological Sciences, University of Leeds, Leeds, United Kingdom; ^2^Living Systems Institute, University of Exeter, Exeter, United Kingdom; ^3^Faculty of Medical and Health Sciences, University of Auckland, Auckland, New Zealand; ^4^Department of Biosciences and Bioengineering, Indian Institute of Technology Bombay, Mumbai, India

**Keywords:** cardiac muscle, couplons, super-resolution, localization microscopy, ryanodine receptors

## Abstract

Remodelling of the membranes and protein clustering patterns during the pathogenesis of cardiomyopathies has renewed the interest in spatial visualisation of these structures in cardiomyocytes. Coincidental emergence of single molecule (super-resolution) imaging and tomographic electron microscopy tools in the last decade have led to a number of new observations on the structural features of the couplons, the primary sites of excitation-contraction coupling in the heart. In particular, super-resolution and tomographic electron micrographs have revised and refined the classical views of the nanoscale geometries of couplons, t-tubules and the organisation of the principal calcium handling proteins in both healthy and failing hearts. These methods have also allowed the visualisation of some features which were too small to be detected with conventional microscopy tools. With new analytical capabilities such as single-protein mapping, *in situ* protein quantification, correlative and live cell imaging we are now observing an unprecedented interest in adapting these research tools across the cardiac biophysical research discipline. In this article, we review the depth of the new insights that have been enabled by these tools toward understanding the structure and function of the cardiac couplon. We outline the major challenges that remain in these experiments and emerging avenues of research which will be enabled by these technologies.

## Background

Historically coined as a name for the focal contacts between the sarcolemma and the sarcoplasmic reticulum (SR) of skeletal muscle (Stern et al., 1997), the term ‘couplon’ in the present day relates more broadly to the nanodomains which encompass the fast calcium (Ca^2+^) signalling mechanisms in striated muscle cells. In cardiac muscle, voltage-dependent inward Ca^2+^ currents (I_Ca_) via L-type Ca^2+^ channels (LCC) at the couplons activate arrays of the giant (>2 MDa) type-2 ryanodine receptor Ca^2+^ channels (RyR2, Cannell et al., 1987) in a Ca^2+^ dependent manner (the mechanism called Ca^2+^-induced Ca^2+^ release or CICR; Fabiato, 1983). Ca^2+^ released from RyRs is the principal activator of cardiomyocyte contraction and is the primary intracellular second messenger in the myocardial excitation-contraction (EC) coupling (see review by Bers, 2002). The large size of cardiac myocytes means that diffusion of the Ca^2+^ released at the cell surface cannot be relied upon for the fast and forceful muscle contraction throughout the cell thickness (Hill, 1949). This problem is circumvented by the tubular invaginations of the sarcolemma which allow couplons to be strategically placed, in a morphology described as ‘dyads,’ mirroring the sarcomeric periodicity of the intracellular organisation of organelles and proteins. Crucial for the cell’s contractile function, this allows highly synchronised cell-wide Ca^2+^ release (Ca^2+^ transient) following an action potential (Cannell et al., 1987). The identification of localised Ca^2+^ release events (Ca^2+^ sparks) (Cheng et al., 1993) as the elementary events of SR Ca^2+^ release consolidated the couplons as the likely structural units of EC coupling. The models of ‘local control’ accounting for the geometrical constraint of the narrow couplon clefts predict the steep dependence of local Ca^2+^ transients on I_Ca_ (Cannell et al., 1995) better than a well-stirred cytoplasm. They emphasise the importance of the couplon architecture, particularly the narrow cytoplasmic cleft space (Soeller and Cannell, 1997), as crucial to its role in EC coupling (Stern, 1992). Adding credence to this point, today, we have a more comprehensive view of the couplon as the hub for the principal Ca^2+^ handling proteins including LCC, RyR and Na^+^/Ca^2+^ exchanger (NCX) as well as numerous regulatory proteins such as calcium/calmodulin-dependent protein kinase II (CaMKII), junctophilin-2 and FK506 binding protein (FKBP). Much of this biophysical understanding comes from microscopy studies spanning over a century, detailing the fine ultrastructure and the mutual arrangement of EC coupling proteins. See the historic account by Franzini-Armstrong (2018a,b) on how this understanding was developed in previous decades.

## Impressions of Couplon Structure From Conventional Microscopies

A decade ago, our view of the cardiac muscle ultrastructure, particularly relating to cardiac couplons, was primarily defined by high quality thin-section (transmission electron microscopy; TEM) and scanning electron microscopy (SEM). The regularly spaced ‘feet’ morphologies in TEM (Flucher and Franzini-Armstrong, 1996; Franzini-Armstrong et al., 1999) and the freeze fractures of sarcolemmal membranes (Sun et al., 1995) were the primary view of couplons. From skeletal muscle EMs, ‘feet’ were quickly identified as individual RyRs (Ferguson et al., 1984); particles and membrane indentations on freeze-fracture SEM images were deduced to be LCCs and the imprints of RyRs (Sun et al., 1995). However, translating the microscopy data of couplons toward understanding the biophysics of Ca^2+^ signalling at the couplon has required a quantitative approach to their imaging and spatial analysis. Capturing the couplon’s three-dimensional (3D) architecture from early EM data involved extensive imaging experience and certain assumptions on the geometries of the cellular compartments. For example, counting RyRs within couplons required awareness of the overall orientation of t-tubules and approximation of couplon circularity and symmetry (Franzini-Armstrong et al., 1999). As discussed by Franzini-Armstrong (2010), detection of ‘feet’ may require alignment of the RyR rows with the microscope axis in TEM. Complementing the EM data, a number of optical (predominantly confocal and widefield) experiments coupled with advanced image analysis (e.g., deconvolution) defined the working model of the couplon structure by the end of the first decade of the 2000s (Scriven et al., 2000; Scriven et al., 2005; Chen-Izu et al., 2006; Soeller et al., 2007; Jayasinghe et al., 2009). From these data, RyR2 clusters associated with cardiac couplons were estimated to typically contain ≥ 100 receptors (Franzini-Armstrong et al., 1999; Soeller et al., 2007). Some co-localisation analyses from immunofluorescence micrographs had demonstrated a very high mutual spatial association between the LCC and RyR consistently in normal cardiomyocytes (Sun et al., 1995; Scriven et al., 2000). These studies reinforced the idea of close mutual alignment between these two complexes, hypothesised based on freeze-fracture SEMs (Sun et al., 1995). Despite some disagreement (Scriven et al., 2000), a moderately strong co-localisation of NCX with the couplon (Jayasinghe et al., 2009) had been confirmed by electrophysiological and *in silico* studies (Lines et al., 2006). However, it is important to note that differences remain between the findings by different research groups that may be attributable to differences in methodology (e.g., fixation, antibody probes, image analysis protocols). This is discussed further in section 9.

**FIGURE 1:**
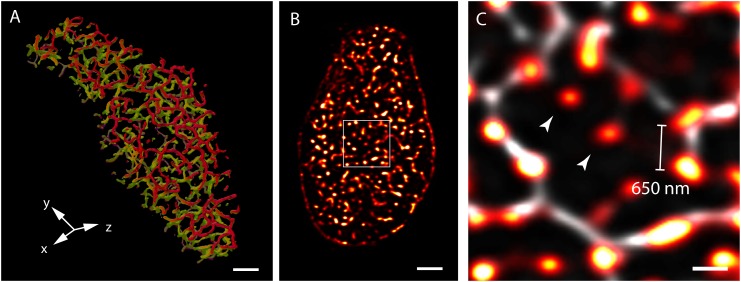
View of the t-system and couplons of rat ventricular myocytes. **(A)** An isosurface reconstruction of the t-tubular network in a myocyte imaged with confocal microscopy in transverse orientation. The regions coloured in red and green respectively are tubules at two adjacent Z-discs; tubules extending between the Z-discs are coloured in purple. **(B)** A transverse view of punctate RyR staining in a myocyte visualised with high resolution confocal imaging; **(C)** Magnified view of the RyR clusters (red hot) of the region demarcated in **(B)**, overlaid with the corresponding t-tubule staining (grey). The typical distance between neighbouring clusters detected with this method was ∼650 nm. Examples of ‘non-junctional’ clusters which did not align with the t-tubules are noted with arrowheads. Scale bars, **(A,B)**: 2 μm, **(C)**: 500 nm. All data re-rendered from Jayasinghe et al. (2009).

Three-dimensional (3D) confocal and multiphoton imaging had revealed the t-tubules to be a dense network with interconnectivity both transversely and longitudinally (Soeller and Cannell, 1999; Jayasinghe et al., 2009) (Figure 1A). An optimised approach to confocal imaging (Chen-Izu et al., 2006), had further revealed couplons, reported by clustered RyR were organised throughout the entire transverse aspect of the Z-discs, much closer to each other (∼600–700 nm) than the previously assumed sarcomeric spacings (∼1.8 μm; Chen-Izu et al., 2006; Soeller et al., 2007; Figures 1B,C). These observations, together with the demonstration of non-planar arrangement of the z-lines at the transverse plane of cardiomyocytes (Soeller et al., 2009; Jayasinghe et al., 2010) led to a series of geometrically realistic simulations of spontaneous propagating Ca^2+^ release (Ca^2+^ waves) throughout the volumes of myocytes (Izu et al., 2006; Soeller et al., 2009; Li et al., 2010). These simulations reinforced the idea that the spatial organisation of couplons plays a vital role in the cell wide Ca^2+^ release properties and emphasised the need to develop models of myocyte EC coupling based on experimentally determined geometries rather than stylised volumes. However, not all of the RyR clusters were found to be associated with ‘couplons’ with the expected co-localisation with the t-tubules. Approximately 15% of RyR clusters in rat ventricular myocytes (Jayasinghe et al., 2009) (Figure 1C) and a larger proportion in rabbit (Sachse et al., 2009) and human (Jayasinghe I. et al., 2012) ventricular myocytes were found to be non-junctional, based on high-resolution 3D confocal image data.

## Resurgence in Ultrastructural Analysis of Failing Myocytes

Confocal microscopy, in particular, played a central role in the pioneering observations of the correlation between dysfunctional intracellular Ca^2+^ release and remodelling of the t-tubules in both animal and human heart pathologies (Gomez et al., 1997; Balijepalli et al., 2003; Louch et al., 2004). The types of remodelling commonly observed through fluorescence imaging included loss of t-tubules in large cytoplasmic regions (Balijepalli et al., 2003; Louch et al., 2004; Crossman et al., 2011), relative increase in the longitudinal t-tubules compared to transverse tubules (Song et al., 2006; Wei et al., 2010), oblique tubules which departed from the z-line locations (Cannell et al., 2006; Crossman et al., 2011, 2015b), increase in the frequency of highly dilated tubules (Crossman et al., 2017) or sheet-like t-tubules (Seidel et al., 2017) (also see section 6 below) in animal and human cardiomyopathies. In the initial confocal-based studies, Song et al. (2006) reported the surprising observation that the highly periodic sarcomeric organisation of RyR clusters appeared undisturbed in confocal micrographs of failing cardiomyocytes from spontaneously hypertensive rats (SHRs), whilst the t-tubule network appeared aberrant and heterogeneous. These observations were the basis of the view that the ensuing loss of local control of these ‘orphaned’ RyR clusters was, at least in part, responsible for the lack of synchrony of Ca^2+^ release throughout the cells [analogous to that seen in ventricular myocytes following osmotically induced detubulation (Brette et al., 2002)]. Any changes to the SR or RyR clusters were not apparent in confocal studies. There was, however, a striking change in the co-localisation between LCCs and RyR as well as structural proteins of the couplon (e.g., junctophilin-2; JPH2) in the cells exhibiting t-tubule remodelling, examined with confocal microscopy (Song et al., 2006; van Oort et al., 2011; Crossman et al., 2015b).

## Limitations in Diffraction-Limited Imaging and Resurging Interest in Couplon Structure

**FIGURE 2:**
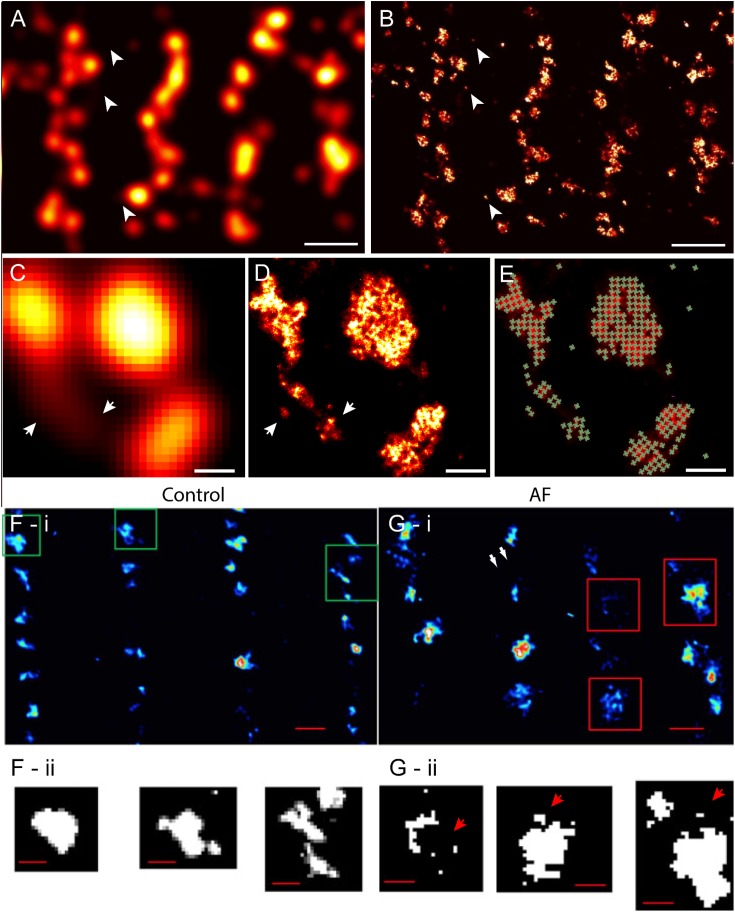
Improved visualisation and analysis of clustered RyR organisation in peripheral couplons of rat ventricular myocytes with dSTORM super-resolution. **(A)** RyR labelling near the surface of a myocyte in diffraction-limited view of RyR clusters, many of which are oblong or elongated in shape. **(B)** The dSTORM image corresponding to the region shown in **(A)**. **(C,D)** Magnified views of matching diffraction-limited and dSTORM images from a few peripheral couplons. Note the small clusters and likely unitary RyRs [indicated in **(A–D)** by arrowheads] are undetectable in the diffraction-limited data. **(E)** To quantify cluster sizes (in RyRs/cluster), quasi-crystalline 30 × 30 nm assembly of RyRs in the regions of labelling was assumed. **(F-i)** Macquaide et al. (2015) compared deconvolved STED images of RyR labelling of healthy sheep atrial myocytes (control) with **(G-i)** RyR labelling in atrial myocytes of a sheep model of atrial fibrillation (AF). Compared to control **(F-ii),** the AF myocytes **(G-ii)** consisted of a higher frequency of smaller RyR cluster (arrows) and a smaller inter-cluster spacings, as illustrated by the magnified views of the clusters outlined in **(F,G-i)**. Scale bars, **(A,B)**: 1 μm, **(C–E)**: 150 nm, **(F,G-i)**: 500 nm, **(F,G-ii)**: 200 nm. **(F,G)** Adapted with permission from Macquaide et al. (2015).

Despite these advances in confocal and similar microscopies, the spatial sensitivity (i.e., resolution) achievable in visualising fine structure of couplons remained limited, by the diffraction of light, to approximately half of the wavelength (i.e., ∼250 nm in optimal imaging conditions) (Abbe, 1873). The typical sizes of cardiac couplons observed in EMs were close to this so-called ‘diffraction limit in resolution’; hence, analysis and interpretation of such optical data were far from straightforward. RyR labelling in putative single couplons often appeared as smooth punctate labelling densities with variable intensities (Soeller et al., 2007) (Figures 1C, 2A). In confocal and total internal reflection fluorescence (TIRF) micrographs, some clusters appeared elongated (Jayasinghe et al., 2009) (see example in Figure 2A). These observations were, at the time, strong indicators that couplons are likely to be of diverse shapes and sizes which could be better studied with an imaging modality with superior resolution. This lack of resolution in existing techniques also posed uncertainty in segmenting images for cluster size and co-localisation analyses (Scriven et al., 2000; Jayasinghe et al., 2009), and could lead to an over-estimation of the spatial overlap of proteins such as LCC and RyR (Scriven et al., 2010; Manfra et al., 2018), raising some concern over contrasting measurements made by independent laboratories. The approach of imaging cardiomyocytes in vertical orientation (achieved by embedding cells in agarose gels; Chen-Izu et al., 2006) offered a modest improvement in the achieved resolution to this end (Jayasinghe et al., 2009; Scriven et al., 2010). Despite this improvement, optical techniques such as confocal microscopy always involved a sensitivity limit such that only t-tubules, couplons or other structures which exceeded a critical size and/or fluorescence labelling density were detectable above background noise (Soeller et al., 2007; Hou et al., 2015).

## Advent of Super-Resolution Optical Microscopy and a Fresh Look at the Couplon

With the demonstration of photoactivated localisation microscopy (PALM, Betzig et al., 2006; Hess et al., 2006) and stochastic optical reconstruction microscopy (STORM, Rust et al., 2006), which are collectively known as ‘single molecule localisation microscopy (SMLM),’ a path was opened for imaging sub-cellular components which were, in size, below the ‘diffraction-limited’ conventional confocal resolution. New and exciting images that were enabled by SMLM included optically resolved focal adhesions (Betzig et al., 2006), mitochondria, endoplasmic reticulum (Shim et al., 2012), caveolae (Gambin et al., 2013), cytoskeletal microtubules (Ries et al., 2012), and actin (Xu et al., 2013). Encouragingly, it also showed great promise in resolving small assemblies of proteins such as the nuclear pore complexes (Loschberger et al., 2012), cell surface receptors, their mobility and clusters (Williamson et al., 2011). All of these spatial features were well beyond the sensitivity and resolution of the conventional optical microscopes.

SMLM, as a principle, relies on the detection and nanometre-scale localisation of fluorescent dye molecules within sample, a subset at a time, until a near-complete map of the dye molecule distribution has been obtained. In common approaches such as PALM or STORM, this is achieved by photoactivating and photo-chemical switching of the fluorescent dye molecules within the sample respectively. The latter particularly relies on the redox chemistry of the microenvironment of the fluorophores to stochastically switch the fluorophores between fluorescent and dark states to observe spatially isolated fluorescent molecules (see detailed review by Sauer and Heilemann, 2017). Long (>30 min) time series of image frame data are recorded and often analysed live to determine the centroid of each fluorescent event corresponding to a single photoactivated/switched fluorescent molecule. Maps of the fluorophore positions compiled over such an image series or density-encoded greyscale images from this type of experiments regularly offer spatial resolution of ∼ 30–50 nm. This near10-fold improvement in the achievable optical resolution was unprecedented and can be anecdotally likened to the difference between being legally blind and gaining twenty-twenty vision^1^. Along with the large improvement in the achievable resolution, SMLM image data also offered an unprecedented detection sensitivity, under suitable conditions approaching single antibodies or marker molecules. With suitably specific markers carefully acquired SMLM images generally involve minimal background signal or noise which has made image analysis, particularly segmentation, much more straightforward than in conventional optical image data.

An important catalyst for the successful uptake of SMLM for studying cardiac cell structure was that it built on existing labelling technologies (e.g., immunofluorescence or fluorescent fusion proteins), therefore sample preparation was, in comparison to EM, still very convenient. Another highly desirable feature is the extremely high sensitivity in detecting marker molecules to the structures of interest (e.g., microtubules stained with nanobody markers by Mikhaylova et al. (2015) compared to marker detection in immuno-gold EMs which are lower in contrast). These unique features provide SMLM a niche in the repertoire of the cardiac cell biophysicist.

In more recent years, we and others have demonstrated that the SMLM image data, particularly the densities of the marker molecules within a sample and the temporal patterns of localisation, encode the underpinning protein target density, independent of the resolution achieved in the final image (Jungmann et al., 2016; Munro et al., 2016). As a result of this, quantification of fluorescence image data is no longer limited to proportional co-localisation analyses. We are now able to detect changes in the protein density within nanoscale cellular domains (Jayasinghe et al., 2014; Munro et al., 2016), count the absolute number of protein targets within a structure of interest (Jungmann et al., 2016) and even estimate the local stoichiometry of protein association (Jayasinghe et al., 2018). These capabilities can make SMLM a more user-friendly and more sensitive approach to quantifying *local* protein changes in cardiomyocytes compared to traditional *in vitro* techniques of measuring protein levels such as calibrated Western Blots of fractionated myocardial tissues or cell suspensions.

The versatility of earlier versions of SMLM were enhanced by the use of the Highly Inclined and Laminated Optical sheet (HILO) illumination approach (Tokunaga et al., 2008) which allows SMLM to be performed in optically thick samples (e.g., thicker than 10 μm). This is particularly relevant to super-resolution imaging of cardiomyocytes which are among the largest cell types in animals. Combining SMLM and HILO has therefore allowed us and others to examine dyads and t-tubules in both isolated cardiomyocytes (Wong et al., 2013; Fu et al., 2016) and hydrated tissue sections (Hou et al., 2014; Crossman et al., 2015a). Despite being a comparatively low-throughput imaging technique in terms of cell numbers (a typical 2D image requiring 10–60 min of acquisition time), dSTORM image data have traditionally allowed the visualisation of large regions in optically thick samples compared to EM ultra-thin sections that are typically 50–60 nm thin (Franzini-Armstrong, 2010). This meant that SMLM offered the researcher the capacity to perform larger-scale spatial statistics [e.g., 1000s of couplons (Jayasinghe et al., 2018)] with relative ease. The typical fields of view in earlier SMLM experiments were limited primarily by the array sizes of the electron multiplying charge coupled device (EMCCD) cameras which were essential for single molecule detection. Within those constraints, a standard dSTORM experiment allowed the super-resolution mapping of a ∼20 × 20 μm 2D area. More recent development of flatfield illumination techniques coupled with large array cameras now expand the field of view to the millimetre range (Douglass et al., 2016). Applications such as these offer the potential to map EC coupling proteins in whole (or multiple) cells conveniently. To date, the majority of the SMLM studies of cardiac myocytes have come from laboratories using custom-built systems, underscoring its robustness as a relatively inexpensive nanoscale imaging technique.

### Early Visualisation of the Nanoscale RyR Organisation

With the earliest application of SMLM (dSTORM, with an estimated resolution of 30–50 nm) to examine cardiac muscle, we were able to resolve more complex shapes and sizes of peripheral couplons, as reported by the clustering of RyRs near the cell surface (Baddeley et al., 2009) (Figures 2A,B). Subsequently, tissue dSTORM images of transversely sectioned myocytes allowed us to resolve and characterise the RyR clusters throughout the entire transverse depth of the cell (Hou et al., 2015). These two investigations, in tandem, demonstrated that dyadic RyR clusters, located deep within the cell interior, were ∼ 4 times larger than the sub-sarcolemmal clusters. This is a distinction which was not clearly made in previous optical or EM data. The single molecule detection approach maps dye molecules within the sample independently of each other. This departure from the reliance from dense immunofluorescence labelling for optical detection allowed the visualisation of solitary (i.e., non-clustered) RyRs for the first time in cardiac muscle (Baddeley et al., 2009; Hou et al., 2015). The vast majority of regions with RyR labelling, in fact, corresponded to solitary RyRs or small clusters (Figures 2A,B). Comparing the super-resolution images with the diffraction-limited images revealed that many of the smaller RyR clusters were virtually undetectable in the latter (Figures 2C,D). The resolved RyR cluster regions enabled us (Baddeley et al., 2009) and others (Macquaide et al., 2015) to estimate upper bounds of the number of receptors within them. For these estimates, it was hypothesised that *in situ* RyR cluster self-assembly reflected the quasi-crystalline patterns at ∼ 30 nm RyR centre-to-centre positioning seen in *in vitro* studies (Yin et al., 2005) (schematically illustrated in Figures 2D,E). In this analysis, an exponential distribution was observed in the cluster sizes, with a high proportion of clusters consisting of <5 receptors. Strikingly, >80% of the cells’ RyRs appeared to reside within a small minority of clusters, each with a size > 100 receptors. (Hou et al., 2015). As a result of the improved sensitivity in detecting RyR clusters, the observable cluster density had doubled from ∼1 μm^-3^ in confocal data (Soeller et al., 2007) to 2.2 μm^-3^ in dSTORM (Hou et al., 2015). In agreement with this observation, the edge-to-edge distances between RyR clusters, in both sub-sarcolemmal and dyadic couplons, were significantly smaller (mean of 140 nm in dSTORM data of interior RyR clusters compared to ∼670 nm with confocal). These were significant revisions to the existing models of RyR distribution within cardiomyocytes and a proposal of a new unifying model of the couplon function (Xie et al., 2010). Given predictions that [Ca^2+^]_i_ within a radial distance of 100 nm outside of the couplon is likely to be elevated to micromolar concentrations (Soeller and Cannell, 1997; Sobie et al., 2006), it was hypothesised that RyR clusters located within a similar edge-to-edge distance are likely to co-activate as a ‘functional super-cluster.’ It meant that 2–6 neighbouring clusters (mean of ∼3.4) within a ‘super-cluster,’ even if not coupled individually to the sarcolemmal triggers, could be recruited rapidly in a ‘triggered saltatory’ fashion. Whether the super-clusters are likely components of the same dyad structure, is yet unresolved. This is due to the lack of markers which allow reliable and independent visualisation of the SR membranes with SMLM. However, the visual analysis performed by Hou et al. (2015) showed that all sub-clusters within super-cluster groupings in the cell interior commonly aligned strongly with the same segment of t-tubule membrane. This supported the idea that an underpinning structural template (e.g., a shared SR terminus) may determine the high mutual proximities between these RyR arrays making up a super-cluster. Whilst this is yet to be confirmed, such an arrangement would have significant ramifications to the way parts of the super-cluster are recruited, the levels of luminal SR [Ca^2+^] seen by each of them and how they may participate in propagating (Izu et al., 2006) or late (Fowler et al., 2018) Ca^2+^ release events. Sub-clusters which are readily recruited by other sub-clusters belonging to the same super-cluster could also create greater redundancy in the LCC/RyR coupling required for optimal Ca^2+^ release synchronisation and maintain the Ca^2+^ contributions from RyR clusters which are seemingly uncoupled from LCC.

### Super-Resolution Insights Into t-Tubule Structure and Accessory Proteins

With renewed focus over the last two decades on t-tubule remodelling coinciding with pathology, SMLM has presented an imaging modality that is superior to conventional optical microscopies for probing the nanometre-scale events which may underpin it. Common t-tubule labelling methods which included antibodies, membrane impermeable dextrans (reporting tubule volume) and membrane dyes used for confocal imaging were broadly compatible with SMLM and other super-resolution techniques. This encouraged a number of groups to promptly adapt super-resolution imaging for t-tubule visualisation. Wagner et al. (2012) used a non-SMLM super-resolution technique known as Stimulated Emission Depletion (STED) microscopy to visualise the topology of the t-tubular membrane in living ventricular myocytes stained with a lipophilic membrane dye. More recently, we characterised the morphological differences between the t-tubular systems in a range of mammalian species (Jayasinghe et al., 2015). We demonstrated that differences in the diameters and the degree of tubule branching was clearly observable between the species. Brandenburg et al. (2016) have used super-resolution STED to reveal the intricate features of a poorly ordered tubular system in human and mouse atrial myocytes which form a series of axial couplons deep in the cell interiors. In the absence of a highly organised t-tubular systems, the combined STED and confocal data led them to propose that these axial couplons in atrial myocytes containing hyperphosphorylated RyR clusters can act as ‘super-hubs’ which relay excitation to the majority of the surrounding RyR cluster which are non-junctional. Among the notable features of t-tubule structures first to be characterised with SMLM, is the tubule dilatations which were observed in two-colour dSTORM images of murine t-tubules (stained for caveolin-3) and RyR (Wong et al., 2013). These tubule dilatations were then confirmed with 3D EM tomograms of mice in the same study and reported later as a feature which may be lost during t-tubule remodelling and the loss of BIN-1 from the couplon coinciding with pathology such as arrhythmia (Hong et al., 2014). Local variations in the t-tubule diameters were likely to be underestimated in confocal and TEM data but were demonstrated well in dSTORM images of small mammal t-tubules and tomographic EMs of rat ventricular myocytes (Pinali et al., 2013).

SMLM has played a major role in revealing the localisation of key structural and functional regulators of couplons. One such protein is Junctophillin-2 (JPH2), which is essential for the formation of cardiac couplons (Takeshima et al., 2000) including the maintenance of the local coupling between the SR and sarcolemmal membranes as well as stabilising the RyR openings (van Oort et al., 2011). The dual-colour mapping of the RyR and JPH2, with super-resolution dSTORM was the first visual demonstration of how accessory proteins (e.g., JPH2) were tightly co-clustered within the couplon (Jayasinghe I.D. et al., 2012). The background-free nature of rendered dSTORM images also allowed robust segmentation of couplon images and estimation of protein co-localisation at an accuracy that was not afforded by previous diffraction-limited imaging data (e.g., Scriven et al., 2000; Jayasinghe et al., 2009). Further to this, dSTORM was instrumental in elucidating the dual structural and regulatory roles of JPH2. The examination of a mouse model overexpressing JPH2 with quantitative dSTORM revealed a larger and rounder RyR cluster morphology but paradoxically resulted in little change in the Ca^2+^ spark properties. Furthermore, only a modest increase in apparent co-localisation between RyR and JPH2 was observed (Munro et al., 2016). A carefully controlled analysis of the relative *densities* of localised RyR and JPH2 markers within each cluster revealed that (i) the larger RyR cluster size was the likely result of a higher local JPH2 density and (ii) despite the approximately unchanged number of RyRs in the clusters, the lower spontaneous Ca^2+^ spark frequency observed in the transgenic animals is the likely result of the additional inhibitory effect imparted on the RyRs by the additional JPH2s.

## Utility of Nanoscale Resolution for Studying Couplon Remodelling

Pathological remodelling of ventricular myocytes was observed over a decade before the development of super-resolution techniques. In a large majority of the studies examining cellular structure in disease, t-tubule remodelling is a dominant feature [see Guo et al. (2013) for a comprehensive list of studies]. Super-resolution microscopy and other nanoscale imaging methods have been useful tools for characterising the finer features of cellular remodelling and probing possible mechanisms. For example, where confocal microscopy was unable to detect any changes in the SR structure, tomographic EM has demonstrated widespread remodelling of the SR in step with local remodelling of t-tubules and mitochondria (Pinali et al., 2013) in heart failure. Despite the previous confocal studies reporting unaltered RyR arrays, deconvolved STED microscopy super-resolution images examining these in atrial myocytes from a sheep model of atrial fibrillation has shown a fragmentation, a reduction of the average cluster-to-cluster distances and greater longitudinal extents of the clusters (Macquaide et al., 2015) (Figures 2F,G). Their computational models predict that these redistributions and remodelling features could explain higher spontaneous Ca^2+^ spark rates and easier propagation of Ca^2+^ waves.

Super-resolution has also been useful for characterising finer features of t-tubule and couplon remodelling as well as probing the underpinning mechanisms. STED image data showing the local *changes* of t-tubule diameter (in the order of 10–25 nm) and orientations in living ventricular myocytes following myocardial infarction (Wagner et al., 2012) were the first to report nanometre-scale remodelling as a feature of this pathology in rat hearts. A number of mechanisms of t-tubule remodelling during pathology that are intrinsic to the myocytes have been characterised. These include the downregulation or cleavage of JPH2 (see review Beavers et al., 2014) and loss of expression of proteins involved in t-tubule biogenesis and maintenance such as bridging integrator-1 (BIN1) (Lyon et al., 2012) and Mitsugumin-29 (MG29) (Correll et al., 2017). In two studies based on a BIN1 deletion mutant mouse model, the authors used tomographic EM and STORM images to suggest that BIN1 expression was essential to developing a complex (folded) membrane topology and functional coupling between LCC and RyR (Hong et al., 2014; Fu et al., 2016). They hypothesised that the loss of this topology, in step with the downregulation of BIN1 expression during failure, leads to a widening of the couplon cleft and diminishing local control of EC coupling. The nanoscale 3D resolution offered by tomographic EM was pivotal to this report. Whilst their analysis consisted of a limited number of example datasets and the folded membrane topology has not been reported elsewhere, it is possible that this t-tubule morphology is either related or equivalent to the junctional dilatation of t-tubules observed previously with dSTORM and EM tomography (Wong et al., 2013). In the cases of JPH2, BIN1 and MG29, visual analysis of t-tubules and couplons in murine models with altered expression of these proteins has been a major strategy of studying mechanisms. In each case, compensatory overexpression of the protein has shown encouraging results in restoring t-tubule structure, or at least function. In each of these avenues, the nanoscale resolution achieved with super-resolution or modern tomographic EM has been essential.

**FIGURE 3:**
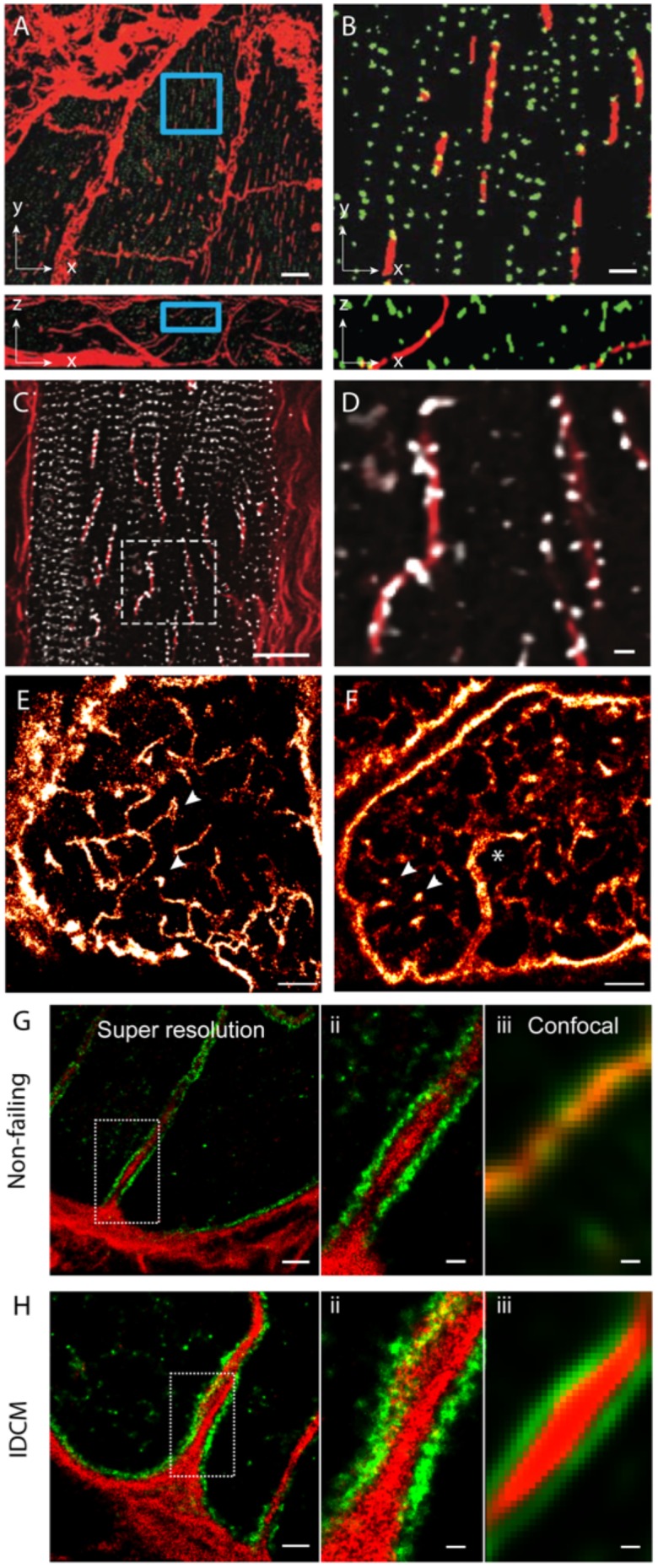
Dilatation of t-tubules in disease and ageing. **(A)** Dilated sheet-like tubules (red) were observed in x-y (upper) and x-z view (lower) by Seidel et al. (2017) in ventricular tissues of patients with chronic heart failure. **(B)** Magnified views illustrate how a subset of RyR clusters (green) appeared to align with these sheet-like tubules (red). **(C)** Similar sheet-like tubules were observed by us in confocal micrographs of ventricular muscle tissue from a human patient with end-stage idiopathic dilated cardiomyopathy (IDCM). **(D)** Magnified view illustrates how a majority of local RyR clusters (grey) are re-arranged along the sheet-like tubules (extending longitudinally, shown in red) instead of maintaining the sarcomeric pattern (transverse). **(E,F)** Compared, are dSTORM images from transverse tissue sections of ventricular cells of young adult (9-weeks old) and aged (100 weeks) stained for t-tubule marker Caveolin-3, respectively. Whilst local dilatations or pockets were observed in the t-tubules in both age groups (arrowheads), macro-tubules (typically > 300 nm in diameter; asterisk in **D**) were observable. **(G,H-i)** Super resolution of both normal and IDCM t-tubules revealed Collagen-VI (red) encased within the tubule lumina whilst dystrophin (green) lined the tubules (magnified view shown in **G,H-ii**). **(G,H-iii)** Illustrate how the limited resolution in the equivalent confocal data fail to reveal this spatial feature in non-dilated t-tubules (in the non failing myocardium). Scale bars: **(A)**: 10 μm, **(B)**: 2 μm, **(C):** 15 μm, **(D):** 2 μm, **(E,F)**: 2 μm. **(G-i,Hi)**: 1 μm, **(G-ii,iii,H-ii,iii)**: 250 nm. **(A,B)** Adapted from Seidel et al. (2017) with permission; **(E,F)** from Crossman et al. (2017) with permission.

In addition to the above features, a handful of new observations of couplon and cellular remodelling now call for further investigation with the use of nanoscale imaging methods. Among the more recently observed phenomena of t-tubule remodelling, we underscore the ‘t-sheets,’ flattened invaginations of the sarcolemma which form longitudinal compartments spanning many sarcomeres. Seidel et al. (2017) have observed such ‘t-sheets’ in ventricular tissues of patients with chronic heart failure. These structures, visualised with 3D confocal microscopy, coincided with an apparent physical and functional reorganisation of a subset of nearby RyR clusters (Figures 3A,B). In tissues that we have examined from human patients with idiopathic dilated cardiomyopathy, we see that a larger proportion of RyR clusters are re-arranged to align with the sheet-like tubules whilst their z-line localisation is lost in the nearby regions of the cytoplasm (Figures 3C,D). This point of difference between our observations and those reported Seidel et al. (2017) at the very least, may underscore different severities of the cellular remodelling pathology associated with heart failure. Alternatively, it could indicate different aetiologies which have not been understood yet. Pinali et al. (2015) have observed ‘super-tubules’ in confocal micrographs and EM tomograms of ischemic border zone tissues following myocardial infarction. In dSTORM tissue imaging, we have observed analogous macro-tubules in a small subset of ventricular myocytes in aged (70–110 week old) mice which display normal cardiac function (Figures 3E,F). Whilst computer modelling has predicted that such structures are the likely result of fused t-tubules, the molecular mechanisms, the time course and the consequences to the nanoscale structure of couplons is yet to be investigated. Our recent work which utilised multicolour dSTORM was able to shed light onto one of the likely mechanisms underpinning t-tubule remodelling, particularly dilatation in heart failure (idiopathic dilated cardiomyopathies; IDCM; Figures 3G,H). It appeared that the excessive deposition of collagen VI within the lumina of t-tubules driven by local fibroblasts and the direct interactions between collagen VI and dystrophin-associated glycoprotein complexes resident on the remodelling t-tubular membrane can impart t-tubule membrane remodelling observed in IDCM. It is noteworthy that confocal data of the same samples lacked the resolution to identify the organisation of the intra-tubular collagen VI, particularly in the non-failing heart. This underscores how the added resolution of dSTORM was instrumental for this mechanistic observation of t-tubule remodelling.

## Models of Couplon Function Informed by Recent Nanoscale Microscopy

The wide ranging RyR cluster sizes observed by Baddeley et al. (2009) with dSTORM were captured in their Monte Carlo model of unconstrained spontaneous cluster assembly to demonstrate how their variable size and shapes can be simulated. Based on simulated and STED-based RyR image data, Walker et al simulated the excitability of the RyRs, given the cluster size, shape and the position of the receptor within the cluster (Walker et al., 2015). By approximating the RyR arrays in each cluster to a well-filled crystalline array, they demonstrated a higher spark fidelity (i.e., probability of evoking a Ca^2+^ spark, given the opening of an LCC) in larger, well-filled RyR clusters. In a supporting simulation, they predicted that a poorly filled RyR cluster would show diminished spark fidelity. However, neither STED nor conventional SMLM techniques offered any insights into variations in the RyR-RyR assembly patterns *within* the clusters. A series of simulations of the effects of fragmented RyR clusters seen in atrial fibrillation build on the hypotheses that smaller clusters may lack allosteric inter-RyR coupling and may be prone to greater Ca^2+^ leak compared to larger clusters in the healthy atrial myocytes (Macquaide et al., 2015). In their model, these two attributes provide possible explanations for the greater spontaneous Ca^2+^ spark and wave probabilities observed in the myocytes from tissues showing atrial fibrillation. Beyond these studies, the number of computational models which capture the nanoscale features of couplons are limited. Whilst tomographic EM data have been exploited to good effect to simulate myoplasmic Ca^2+^ dynamics in the sub-micron scale (Rajagopal et al., 2015; Colman et al., 2017), these models do not directly utilise experimentally determined shapes and sizes of couplons or the positions of RyRs within the couplons. Rather, the couplons are simplified as point sources of Ca^2+^. However, because EM tomograms used thus far have lacked information on the molecular configuration of couplons (e.g., RyR positions), such models have tended to approximate couplons as unitary structures, lacking information on variabilities in cluster sizes, shapes and spacings.

## Advancing the Resolution and Quantitative Utility of SMLM

### Limitations in Contemporary Nanoscale Imaging Techniques

Approaching a decade since the first application of SMLM to study couplons, we have learnt that the resolution achievable with techniques like dSTORM transformed our view of the shapes and mutual positions of RyR clusters. However, with resolution limited to ∼30–50 nm, dSTORM failed to resolve the receptor arrangement pattern within the individual couplon architecture. In particular, it has become clear that the pattern of signal across the extent of RyR clusters observed with dSTORM (see, for example, the enlarged cluster in Figure 1D) results almost exclusively from stochastic dye switching and exhibits little correlation with the distribution of receptors across the cluster [see simulation in Supplementary Figure 2A in Jayasinghe et al. (2018)]. ‘Counting’ of the number of RyRs in each cluster was therefore based rigidly on the assumption that RyR arrays were well-filled quasi-crystalline arrays with receptor assembly with a centre-to-centre distance of ∼30 nm. This approximation was supported by the regularity of the ‘feet’ spacing in early thin-section TEMs of cardiac couplons and the SEMs of RyR *in vitro* self-assembly observed by Yin and Lai (2000) and Yin et al. (2005). The emerging tomographic EM data from mouse and rat ventricular myocytes in 2009 argued, however, that RyR arrays are unlikely to be uniformly filled (Asghari et al., 2009; Hayashi et al., 2009). It was also not clear whether the area occupied by RyR labelling in dSTORM images was an accurate representation of the couplon geometry, due to the lack of an independent marker for the SR, particularly terminal SR, membranes. EM tomography data appeared to argue that the couplons were a larger structure than reported by the positions of RyRs (Asghari et al., 2009; Hayashi et al., 2009). However, this observation was subject to the manual segmentation approaches that were necessary for reconstructing RyR arrays in the image data which, at the time, lacked an RyR-specific contrast mechanism. Tilt-EM tomograms of RyR in rat ventricular couplons published more recently revealed more convincingly that receptor arrangement can be non-uniform and that they can acutely re-organise into a quasi-crystalline pattern upon altered receptor phosphorylation and changes in free [Mg^2+^]_i_ (Asghari et al., 2014). However, detection of couplons with this method was essentially manual and therefore favours larger couplons (e.g., ∼21 RyRs within a single cluster) where an obvious RyR array pattern is visible. There is to date no demonstrated evidence suggesting that the current state of tomographic EM can faithfully detect and segment smaller RyR clusters or solitary RyRs. Whether a free [Mg^2+^]_i_ -dependent re-arrangement of RyRs is present similarly in small RyR clusters (e.g., <9) is therefore not known.

### DNA-PAINT as a Tool to Visualising Couplons at the True Molecular Scale

**FIGURE 4:**
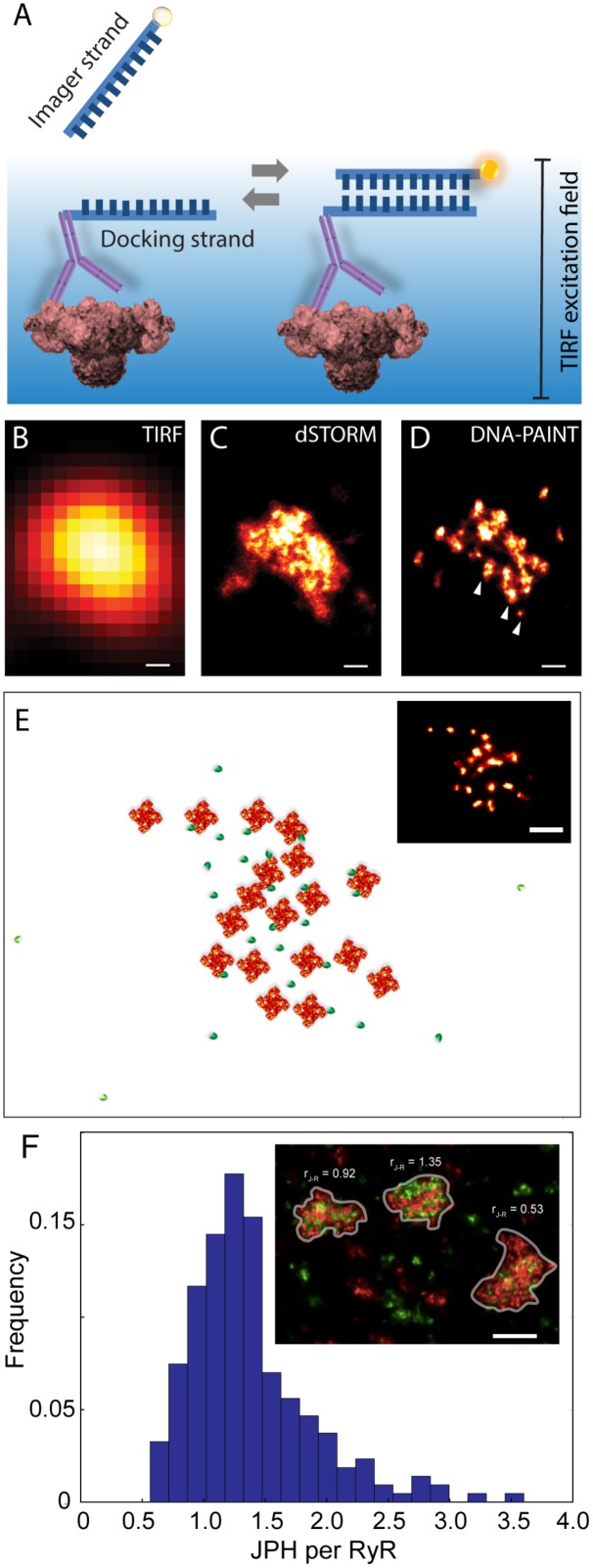
Adaptation of DNA-PAINT to mapping RyR in peripheral couplons of rat cardiomyocytes. **(A)** DNA-PAINT relies on the thermally driven stochastic and reversible binding between a fluorescently labelled ‘imager’ DNA oligo and a ‘docking’ DNA oligo which in this scenario is linked to the anti-RyR antibody. Markers near the cell surface are illuminated with a TIRF excitation field. Shown, is a comparison of **(B)** conventional TIRF, **(C)** dSTORM and **(D)** DNA-PAINT images of an RyR cluster, highlighting the detection of finer punctate labelling densities which are unique to the latter (arrowheads). **(E)** Example of an approximate reconstruction of individual RyR arrangement (red) and hypothesised accessory proteins such as JPH2 (green) based on the RyR positions reported by an experimental DNA-PAINT image of a peripheral couplon (inset). **(F)** Histogram illustrating the distribution of JPH2 to RyR ratios [mean 1.38, mode at 1.25 and a width of 0.5 (standard deviation), *n* = 250 clusters (containing ≥ 15 RyRs)]. Inset illustrates examples of three adjacent couplons exhibiting JPH2/RyR ratios ranging between 0.53 and 1.35 (RyR shown in red and JPH2 in green). Scale bars: **(B–D)**: 50 nm, **(E)**: 100 nm, **(F)**: 250 nm. All panels adapted from Jayasinghe et al. (2018).

An alternative SMLM technique, DNA-PAINT was described in Jungmann et al. (2014). The authors demonstrated its capacity to offer superior resolution in imaging intracellular structures (<10 nm) and greater versatility in multiplexed imaging compared to dSTORM. These two benefits were largely the product of its departure from the photochemical principle which underpinned existing SMLM methods such as dSTORM. The single molecule localisation precision in dSTORM, one of the crucial determinants of resolution, intrinsically depended on the photons which were detectable from each fluorophore photoswitching event (Mortensen et al., 2010) which, in turn, was a function of the redox microenvironment of the sample (Bates et al., 2007). Over the last decade many laboratories around the world, including us, have found it challenging to control or maximise the photon yield to obtain sub-10 nm localisation precision. DNA-PAINT achieves this by complete departure from photochemical switching of fluorophores. The markers in DNA-PAINT are localised by the thermally driven reversible hybridisation of complementary strands of DNA oligonucleotides that leads to transient immobilisation of dye molecules on markers as summarised in Figure 4A. As a result, brighter and longer single molecule events are achieved enabling higher photon yield and hence improved signal-to-noise ratio and marker localisation precision. We have recently used this method for mapping RyRs in peripheral couplons of myocytes at resolution of ∼10–15 nm (Jayasinghe et al., 2018).

At a magnified view, the DNA-PAINT images of RyR distribution reported highly distinctive and highly reproducible ‘punctate’ labelling densities within the cluster area, which were not observed in the dSTORM images (Figures 4B–D). Utilising correlative dSTORM imaging experiments and a target counting algorithm called qPAINT (Jungmann et al., 2016), we were able to confirm that these puncta corresponded to individual RyRs within clusters which were first resolved nearly a decade ago with dSTORM. Their individual positions offered a more realistic view of the *in situ* positioning of RyRs and accessory proteins such as JPH2 in the intact couplon (see Figure 4E for an approximate reconstruction). Exploiting the novel ability to visualise large areas of the cell with sub-10-nanometre resolution, we sampled large numbers (>1000) of RyR clusters to robustly observe that peripheral RyR clusters, on average, contained ∼9 RyRs – a number that is approximately half of that estimated a decade ago with dSTORM (Baddeley et al., 2009). Consistent with the tomographic EM analysis (Asghari et al., 2014), the DNA-PAINT maps of RyR revealed an irregular receptor arrangement which was not consistent with the crystalline array structures observed previously *in vitro* (Yin and Lai, 2000; Yin et al., 2005). However, this may be compatible with the more stochastic RyR-RyR associations (called ‘branched arrays’) predicted by computational modelling based on *in vitro* interactions observed between adjacent receptors (Cabra et al., 2016). Large (>50 nm) gaps which were observed within the RyR clusters in DNA-PAINT image data may be a manifestation of this type of *in situ* cluster assembly. With the superior resolution and robust detection of the RyR positions, DNA-PAINT provided an opportunity to visualise JPH2 co-clustering at the couplons. Based on spatial and target counting analyses, multiplexed DNA-PAINT data revealed that JPH2 is, on average, localised within 50 nm of the centre of RyR channels in a likely bound configuration. It was also observed that the stoichiometry of JPH2:RyR co-clustering varied significantly (between 0.5 and 2.5) in the couplons of the same cell (Figure 4F). Noting that the density of local JPH2 regulates the excitability of RyRs (van Oort et al., 2011; Munro et al., 2016), it has allowed us to propose that the variable co-clustering of regulatory proteins such as JPH2 in couplons could represent a previously unseen mechanism of regulating Ca^2+^ release function in a given cytoplasmic locality.

## Current Understanding of Couplon Structure-Function Relationship and Future Challenges

**FIGURE 5:**
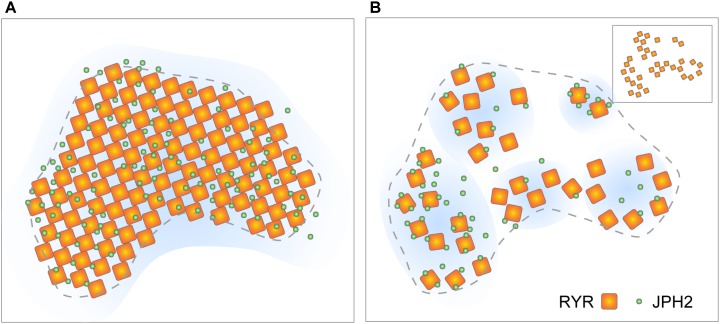
Evolving view of RyR arrangement within couplons. **(A)** The classic model of quasi-crystalline arrangement of RyR and near-uniform co-clustering with JPH2 within well-filled couplon sections. **(B)** Revised model based on tomographic EM and DNA-PAINT data proposing looser arrangement of RyRs and variable co-clustering with JPH. Inset illustrates the scenario where a diagonal (‘checkerboard’) arrangement of RyRs could be achieved even in the loosely arranged clusters. Adapted from Jayasinghe et al. (2018).

With the new molecular-scale visualisations, both with the latest SMLM and the advancing tomographic EM technologies, it is our observation that couplon structure appears to be less stereotypic than previously assumed (i.e., RyR arrays are less crystalline and less well filled; Figures 5A,B). However, it is important to note that quasi-crystalline RyR array patterns [i.e., side-by-side or diagonal/‘checkerboard’ arrangement of neighbouring RyRs, observed by Asghari et al. (2009)] are still compatible with the receptor positions mapped by DNA-PAINT (see Figure 5B inset). Assessing the DNA-PAINT data, it is unlikely to be orchestrated by direct RyR-RyR contact via the SPRY1/P1 domain interactions. The fact that the patterns are sensitive to [Mg^2+^]_i_ and phosphorylation state (Asghari et al., 2009) could support the idea that the RyR array patterns are organised by accessory proteins resident within the couplon. These features, in fact, reveal new dynamic aspects of the couplon structure. More recent reports based on live cell confocal imaging of myocytes expressing RyR-GFP fusion proteins (Hiess et al., 2018) demonstrate that large RyR clusters located at the cell periphery might be subject to acute turnover or mobilisation. Both the simulations based on STED analyses (Walker et al., 2014; Wang et al., 2014) and the JPH2/RyR co-clustering analysis from DNA-PAINT (Wang et al., 2014; Jayasinghe et al., 2018) suggest that couplons are likely to possess heterogeneous excitabilities. They may also possess different likelihoods of Ca^2+^ release termination, depending on the RyR-spacings and the couplon cleft spaces resulting from variable RyR arrangement; however, the current computer models need to explore the RyR array morphologies within their spatial models (Gillespie and Fill, 2013; Laver et al., 2013). Variability in the couplon structure and/or composition may also underly mechanisms giving rise to the spontaneous late Ca^2+^ sparks characterised recently by Fowler et al. (2018) as a potentially arrhythmogenic phenomenon.

### Limitations of the Current Nanoscale Couplon Imaging Experiments

Despite the rapid uptake of SMLM by hundreds of research groups worldwide, the number of groups which have comfortably incorporated this technology into investigations on myocyte, particularly couplon, structure remains limited. This may be because SMLM represents either a considerable financial investment (in off-the-shelf instrumentation) or, alternatively, a skill investment of specialist researchers to harness the open source software tools which are essential for it. However, nearly a decade on from our first experiments, SMLM, particularly DNA-PAINT, remains a robust approach to achieving single-protein level of resolution in imaging couplons. With the demonstration of various applications of DNA-PAINT, particularly in imaging optically thick samples (Auer et al., 2017; Lutz et al., 2018) in 3D imaging protocols (Schueder et al., 2017), it is set to extend our view of the couplon structure well beyond the sub-sarcolemmal nanodomains resolved recently (Jayasinghe et al., 2018). However, it needs to be emphasised that the accuracy of the super-resolution imaging extends only as far as the reliability and availability of probes, particularly antibodies. Whilst a number of studies have used well-characterised antibodies against RyR2, the access to a reliable LCC antibody remains limited to a handful of research groups. Repeated (and mostly unpublished) immunolabelling data of LCC by a number of other groups underscores the currently limited opportunity to visualise the local couplings of RyR2 and LCC at the single-protein level of resolution that is now available. In our view, this challenge calls for a multi-partisan approach to comparing antibody (or alternative specific) probes and tissue/cell preparation protocols. From the data that has been published thus far, DNA-PAINT analyses appear to strongly complement the tomographic EM approaches that have been used to visualise the arrangement of single RyRs (Asghari et al., 2009, 2014). Correlative SMLM/EM protocols (e.g., Löschberger et al., 2014), as laborious and time consuming as they are, may capture the best of both nanoscale approaches. Incorporation of these spatial features into geometrically realistic computer models will continue to provide crucial insights into how this nanostructure can determine function. However, these models would benefit from live cell-super resolution microscopy measurements. Transgenic mice expressing fluorescent protein-fused RyRs that have recently been made (Hiess et al., 2018; Hou et al., 2018) could provide a unique opportunity to visualise these proteins at the nanoscale in the living cell.

## Ethics Statement

Figures 3C,D includes original images recorded from a human cardiac tissue sample of a failing heart, obtained from the Auckland City Hospital transplant program. All human tissue was obtained with written and informed consent from transplant recipients or families of organ donors in accordance to the declaration of Helsinki, institutional guidelines as approved by the Health and Disability Ethics Committee of New Zealand (NTY/05/08/050/AM05). Figures 3E,F include original images acquired from heart tissue of C57BL/6 mice. These experiments were done according to a protocol approved by the Animal Ethics Approval Committee of the University of Exeter (reference: EMPS-2014-1). All other data are re-productions of data that were published previously, as per citations.

## Author Contributions

IJ, DC, and CS conceived the idea. IJ, AC, OdL, and SS acquired or re-analysed the data. IJ, DC, and CS wrote the paper.

## Conflict of Interest Statement

The authors declare that the research was conducted in the absence of any commercial or financial relationships that could be construed as a potential conflict of interest.

## Footnotes

1 We refer to visual acuity which is a measure of the sharpness or clarity of vision. Based on the Hermann Snellen charts, visual acuity measurement of 20/20 relates to matching the resolution of ‘normal’ human vision (i.e. the ability to resolve contours which are 1.75 mm apart from a distance of 20 ft) from 20 ft away. In North America and parts of Europe, the limit for legal blindness is 20/200 which corresponds to a 10-fold reduction in the visual acuity, broadly similar in proportion to the resolution difference between diffraction-limited techniques and SMLM.
